# Association between insulin resistance and left ventricular hypertrophy in asymptomatic, Black, sub-Saharan African, hypertensive patients: a case–control study

**DOI:** 10.1186/s12872-020-01829-y

**Published:** 2021-01-02

**Authors:** Bernard Kianu Phanzu, Aliocha Nkodila Natuhoyila, Eleuthère Kintoki Vita, Jean-René M’Buyamba Kabangu, Benjamin Longo-Mbenza

**Affiliations:** 1 Cardiology Unit, University Hospital of Kinshasa, PO Box 1038, Kinshasa, Democratic Republic of Congo; 2 Centre Médical de Kinshasa (CMK), Kinshasa, Democratic Republic of Congo; 3 Department of Biostatistics, Public Health School, Kinshasa, Democratic Republic of Congo

**Keywords:** Hyperinsulinemia, Insulin resistance, Obesity, Sedentary time, Left ventricular hypertrophy, Left ventricular mass, Hypertension, Black, Sub-saharan african

## Abstract

**Background:**

Conflicting information exists regarding the association between insulin resistance (IR) and left ventricular hypertrophy (LVH). We described the associations between obesity, fasting insulinemia, homeostasis model assessment of insulin resistance (HOMA-IR), and LVH in Black patients with essential hypertension.

**Methods:**

A case–control study was conducted at the Centre Médical de Kinshasa (CMK), the Democratic Republic of the Congo, between January and December 2019. Cases and controls were hypertensive patients with and without LVH, respectively. The relationships between obesity indices, physical inactivity, glucose metabolism and lipid disorder parameters, and LVH were assessed using linear and logistic regression analyses in simple and univariate exploratory analyses, respectively. When differences were observed between LVH and independent variables, the effects of potential confounders were studied through the use of multiple linear regression and in conditional logistic regression in multivariate analyses. The coefficients of determination (R^2^), adjusted odds ratios (aORs), and their 95% confidence intervals (95% CIs) were calculated to determine associations between LVH and the independent variables.

**Results:**

Eighty-eight LVH cases (52 men) were compared against 132 controls (81 men). Variation in left ventricular mass (LVM) could be predicted by the following variables: age (19%), duration of hypertension (31.3%), body mass index (BMI, 44.4%), waist circumference (WC, 42.5%), glycemia (20%), insulinemia (44.8%), and HOMA-IR (43.7%). Hypertension duration, BMI, insulinemia, and HOMA-IR explained 68.3% of LVM variability in the multiple linear regression analysis. In the logistic regression model, obesity increased the risk of LVH by threefold [aOR 2.8; 95% CI (1.06–7.4); p = 0.038], and IR increased the risk of LVH by eightfold [aOR 8.4; 95 (3.7–15.7); p < 0.001].

**Conclusion:**

Obesity and IR appear to be the primary predictors of LVH in Black sub-Saharan African hypertensive patients. The comprehensive management of cardiovascular risk factors should be emphasized, with particular attention paid to obesity and IR. A prospective population-based study of Black sub-Saharan individuals that includes the use of serial imaging remains essential to better understand subclinical LV deterioration over time and to confirm the role played by IR in Black sub-Saharan individuals with hypertension.

## Background

Hypertensive patients with insulin resistance (IR) are at increased risk of cardiovascular events compared with hypertensive patients without IR [1]. Similarly, the presence of hypertension (HTN)-mediated organ damage (HMOD), including left ventricular hypertrophy (LVH), has well-established adverse prognostic significance [2].

IR is classically defined as the impaired biological response of target tissues to insulin stimulation [3]. Gerald M. Reaven’s pioneering works have suggested the existence of a pathophysiological link between IR and almost all known cardiovascular risk factors. Reaven is fondly remembered as the father of IR due to his contributions to our current understanding of the central role played by IR in cardiovascular disease, including the development of the insulin suppression test, which was the first quantitative method introduced to assess insulin-mediated glucose uptake in humans [4]. Using this test, Reaven established the important contributions of IR to human disease, particularly type 2 diabetes [5, 6]. In a non-diabetic patient population, he illustrated the roles played by IR in the development of essential HTN [7]; osmotic balance [8]; sympathetic nervous system stimulation [9]; hypercoagulability [10]; decreased urinary uric acid clearance, with resultant hyperuricemia [11]; increased postprandial lipemia and the accumulation of residual lipoproteins [12]; the occurrence of lipid abnormalities, such as hypertriglyceridemia [13]; low levels of high-density lipoprotein cholesterol (HDL-c) [14]; and a decrease in the diameter of low-density lipoprotein cholesterol (LDL-c) particles [15].

LVH is a type of HMOD and is known to be a full-fledged cardiovascular risk factor associated with poor prognostic value [16–19]. Despite extensive studies, the pathophysiology of cardiac hypertrophy remains poorly understood [20], although both genetic [21, 22] and environmental factors [23, 24] are thought to contribute to the development of this disease. IR is one environmental factor that has been cited as being associated with LVH occurrence [24–26].

However, conflicting information exists regarding the association between IR and LVH in hypertensive patients. We sought to assess this relationship in a hypertensive sub-Saharan Black population.

## Methods

### Study design and setting

The present study was a case–control study conducted at the Centre Médical de Kinshasa (CMK) between January and December 2019. The CMK is a reference clinic that operates according to international standards and norms, with a cardiology unit named “Pôle de Cardiologie” (cardiology center), which is staffed by highly qualified and regularly retrained personnel. The cardiology unit provides cardiovascular explorations, such as Doppler echocardiography, coronary scanning, and cardiopulmonary exercise testing. A cardiovascular rehabilitation unit is also operational at this hospital and represents the only such unit in central Africa.

### Patient selection

Consecutive asymptomatic hypertensive patients, aged 20 years or older, who attended the outpatient clinic of the CMK Pôle de Cardiologie between January and December 2019, were screened for clinical or laboratory evidence of secondary HTN and renal or hepatic disease. Patients in whom a cause of secondary HTN was found, as well as patients diagnosed with renal or hepatic disease, were excluded from this study. All other patients were invited to sign written informed consent forms to participate in this study and underwent cardiac Doppler ultrasound.

Participants with heart disease unrelated to high blood pressure (BP) were excluded from this study. Each participant who met the echocardiographic diagnostic criteria for LVH was matched for sex and age with two hypertensive patients without LVH.

**Fig. 1:**
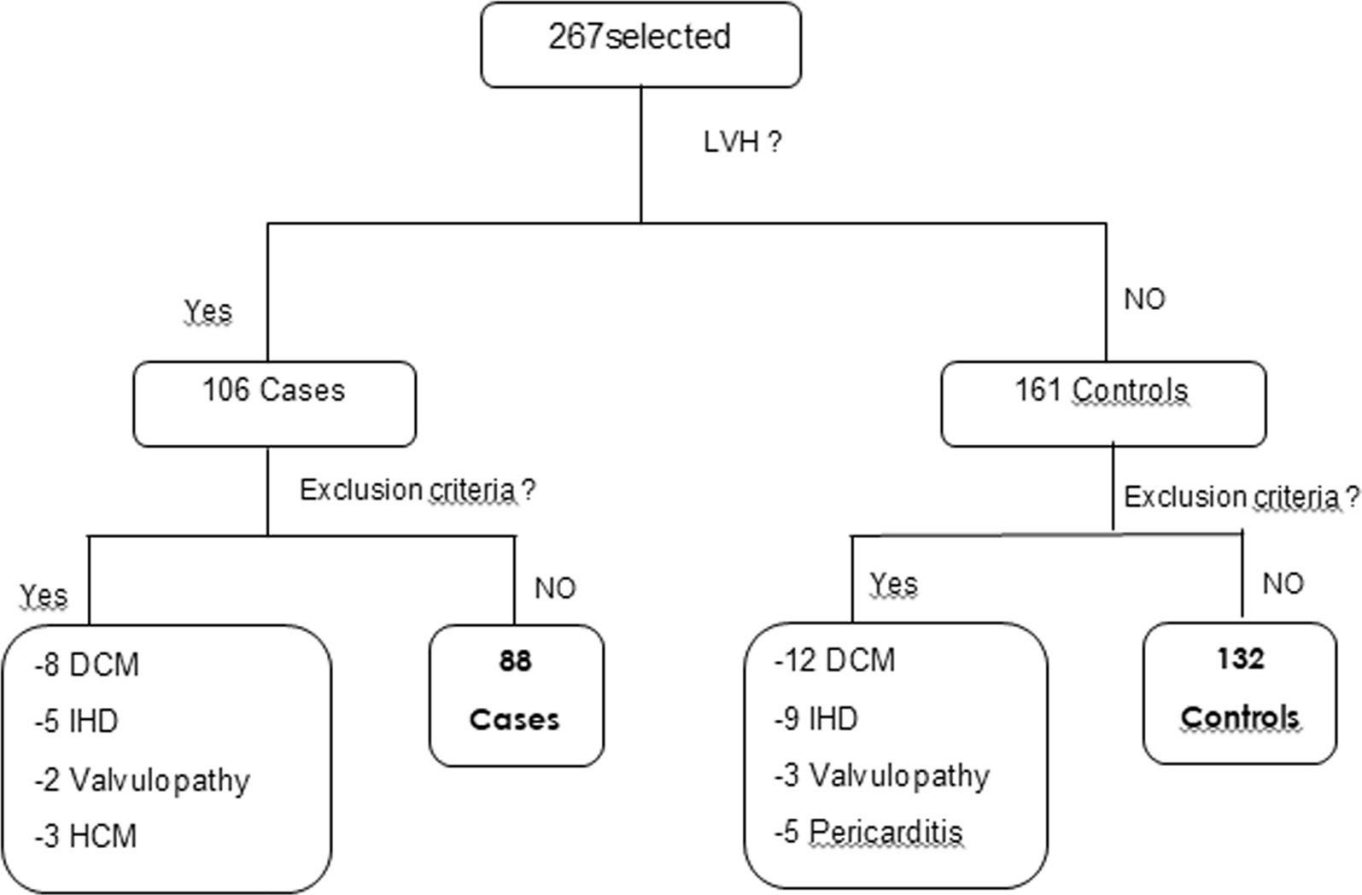
Flow chart summarizing inclusion and exclusion of cases and controls

A total of 267 participants were initially selected to participate in the study, including 106 with LVH and 161 without LVH. Of these, 47 were excluded for various reasons, including: dilated cardiomyopathy in 20 participants (8 with LVH and 12 without LVH); ischemic cardiopathy in 14 participants (5 with LVH and 9 without LVH); significant valvulopathy in 5 participants (2 with LVH and 3 without LVH); pericarditis in 5 participants without LVH; and hypertrophic cardiomyopathy in 3 participants with LVH. Therefore, the final analysis included 220 participants: 88 (40%) with LVH and 132 (60%) without LVH. The flow chart in Fig. 1 summarizes the selection of cases and controls.

### Study procedures

#### Anamnestic data

Anamnestic data were obtained using a standard questionnaire. The anamnesis focused on self-reported age, gender, sedentary behavior, alcohol use and smoking habits, history of diabetes mellitus, and current medication use to treat chronic diseases, especially antihypertensive drugs, anti-diabetic treatments, statins, antiplatelet agents, hypouricemics, oral contraceptives, and hormone replacement therapy. Participants were also asked to report their histories of cardiovascular events, including stroke, ischemic heart disease, heart failure, chronic kidney disease, and cardiovascular surgery.

#### Anthropometric data

The measurement of anthropometric parameters was performed by a final-year medical student who had previously undergone a practical training session for this purpose. Weight and height were measured using a validated electronic weight scale and a wall height gauge, respectively, while the participant was standing, barefoot, and lightly dressed. Body mass index (BMI) was calculated as the ratio of weight (kg) to height squared (m^2^). The waist circumference (WC) and hip circumference (HC) were obtained in cm using a tape measure.

The body surface area (BSA) was calculated using the DuBois formula [27], as follows:

$${\text{BSA }}\left( {{\text{m}}^{{2}} } \right) \, = \, 0.{\text{725 height }}\left( {{\text{cm}}} \right) \, \times \, 0.{\text{425 weight }}\left( {{\text{kg}}} \right) \, \times \, 0.00{718413}.$$

#### Blood pressure

Blood pressure (BP) was measured non-invasively through 24-h ambulatory blood pressure monitoring (ABPM) using a fully automatic recorder (Model TONOPORT V; GE Health care, Freiburg, GERMANY). The recorder was programmed to perform a BP measurement every 15 min during the waking period and every 30 min during sleep. The 24-h average BP was used for these analyses.

#### Echocardiographic data

In all participants, a detailed two-dimensional transthoracic echocardiography was performed by a single certified cardiac sonographer using a commercially available system (Vivid T8, GE Health care, Freiburg, GERMANY) equipped with a 3.5 MHz transducer. Left ventricular measurements were obtained, according to the 2015 American Society of Echocardiography and the European Association of Cardiovascular Imaging updated guidelines for cardiac chamber quantification [28]. Measurements of left ventricular diameter (LVED), interventricular septum thickness (IVS), and posterior wall thickness (PWT) were measured at the end of diastole. Simultaneous ECG was performed to correlate the left ventricular measurements with the cardiac cycle. Left ventricular mass (LVM) was calculated according to the American Society of Echocardiography simplified cubed equation linear method, using the following equation: LVM (grams) = 0.8 × 1.04 × [(LVED + IVS + PWT)^3^ − (LVED)^3^] + 0.6 g, where LVED is the left ventricular end-diastolic diameter, IVS is the interventricular septal thickness, and PWT is the left ventricular posterior wall thickness. Left ventricular mass was indexed against BSA and height [2, 7]. The relative wall thickness (RWT) was calculated as follows: (2 × PWT) / LVED.

In accordance with international recommendations [29], LV diastolic function was assessed from the apical four-chamber view, which included transmitral, pulsed-wave Doppler and mitral annular velocities with tissue Doppler echocardiography. The transmitral peak early (E) and peak late (A) diastolic velocities were recorded. The mitral annular early diastolic velocity (e’) was measured at the lateral mitral annulus using pulsed-wave tissue Doppler in the apical four-chamber view with gains minimized to allow for a clear tissue signal.

#### Laboratory measurements

For all analyses, all participants provided a morning blood sample after an overnight fast. All samples were analyzed at the CMK laboratory. The blood for the determination of serum uric acid, total cholesterol (TC), LDL-c, HDL-c, and triglycerides was collected in a dry tube. The assay used to measure these biological parameters was performed using standard colorimetric methods, and the readings were performed using the HELIOS Epsilon brand colorimetric spectrophotometer (Milwaukee, USA). Glucose was assayed on oxalated plasma according to a colorimetric method using the “BIOLABO” test (France).

Insulinemia was assessed using ethylenediaminetetraacetic acid (EDTA) plasma by enzyme-linked immunosorbent assay (ELISA). The optical density reading was performed on a string read from HUMAREADER HUMAN (Germany).

Assessments of glycated hemoglobin (HbA1c) were performed using EDTA plasma by the electrophoretic method, with HYRYS HYDRASIS from SEBIA (France).

Serum creatinemia was measured by the simple colorimetric Jaffe method. Readings were assessed with a colorimetric spectrophotometer (Spectrum 2100 brand, South Africa).

### Operational definitions

#### Lifestyle data

Sedentary was defined as sitting for more than 7 h a day [30]. Cigarette smoking was defined as regular smoking for at least 30 days preceding the interview date, regardless of the number of cigarettes smoked [31].

Excessive alcohol consumption was defined as drinking more than 2 glasses of beer or its equivalent every day for at least a year [32].

#### Anthropometric parameters

Overweight was defined as a BMI between 25 and 29.9 kg/m^2^ of BSA [33].

Obesity was defined as a BMI equal to or greater than 30 kg/m^2^ of BSA [33]. Abdominal obesity was defined as a WC greater than 102 cm for men and greater than 88 cm for women [33].

#### Bioclinical data

Poor control of arterial HTN was defined as an average systolic BP greater than 130 mmHg or an average diastolic BP greater than 80 mmHg, as assessed by 24-h ABPM [34].

#### Paraclinical data

Diabetes mellitus was defined as a fasting blood glucose level ≥ 10 mmol/L and HbA1c > 7% [35].

Hyperinsulinemia was defined as fasting insulinemia > 90 mmol/L.

IR was defined as HOMA-IR ≥ 2.5 [36].

Dyslipidemia was defined as an HDL-c level of < 1.03 mmol/L for men and < 1.04 mmol/L for women, an LDL-c level ≥ 3.38 mmol/L, a TC level ≥ 5.17 mmol/L, or a triglyceride level ≥ 1.69 mmol/L [37].

The atherogenicity index (AI) was calculated as the TC-to-HDL-c ratio. The AI was considered high when this ratio was greater than 5 [38].

Hyperuricemia was defined as a fasting uric acid level > 420 mmol/L [39].

#### Echographic data

LVH was defined as LVM > 115 g/m^2^ or > 48 g/m^2.7^ for men when indexed to BSA or to height, respectively, and as LVM > 95 g/m^2^ or > 44 g/m^2.7^ for women when indexed to BSA or to height, respectively. Four LV geometric patterns were defined as follows [40]: normal geometry (normal LVM and RWT ≤ 0.42), concentric remodeling (normal LVM and RWT > 0.42), eccentric hypertrophy (LVH and RWT ≤ 0.42), and concentric hypertrophy (LVH and RWT > 0.42).

Three patterns of diastolic dysfunction (DD) were defined according to the E/A ratio, as follows [41, 42]: abnormal relaxation (grade I DD: E/A ratio < 1 and prolonged deceleration time), pseudonormal relaxation (grade II: E/A ratio > 1 and intermediate deceleration time), and restrictive patterns (reversible and irreversible, grades III and IV, respectively; E/A ratio > 2 and shortened deceleration time).

Normal left ventricular filling pressure (LVFP) was defined by an E/e’ ratio < 8 [43]. Elevated LVFP was defined by an E/e’ lateral > 12 [43]

The dilation of the left atrium (LA) was defined as an LA body surface area > 20 cm^2^ [44].

### Statistical analyses

Data are presented as the absolute (n) and relative (%) frequencies for categorical variables and as averages (± standard deviation) for quantitative variables. Paired comparisons between the cases and controls were performed using Pearson’s Chi-square test or Fisher’s Exact test, as appropriate, for categorical variables and using Student’s t-test for continuous variables.

Linear regression was used to determine the predictive factors associated with LVM variations. The following variables were entered in the univariate analysis: parameters of obesity (WC, HC, and BMI), parameters of glucose metabolism (fasting glucose, HbA1c, fasting insulinemia, and HOMA-IR), parameters of lipid metabolism (TC, HDL-c, LDL-c, and triglycerides), parameters of renal function (creatinine and uricemia), parameters of phosphocalcic metabolism (calcium, ionized calcium, and phosphorus). When significant associations were observed between LVM and these independent variables, the effects of potential confounders were studied by adjustment in multiple linear regression.

Simple logistic regression was used to determine which factors were predictive of LVH. The following variables were entered into the univariate analysis: Medical and social history (duration of HTN, cigarette smoking, excessive alcohol consumption, and menopause), sedentary lifestyle, uncontrolled HTN, dyslipidemia, high AI, diabetes mellitus, hyperinsulinemia, hyperuricemia, and IR. When associations were observed between LVH and these independent variables, the effects of potential confounders were studied by adjustment in a conditional logistic regression (multivariate analysis).

The significance threshold retained was p < 0.05. Statistical analyses were performed using XLStat 2020 and SPSS (Statistic Package for Social Sciences) software for Windows, version 24.

## Ethical considerations

This research was conducted in strict compliance with the recommendations of the Helsinki Declaration III. Approval to conduct the study was obtained from the National Health Ethics Committee (No. 219/CNES/BN/PMMF/220). All respondents were debriefed on the results of the study.

## Results

### Characteristics of cases and controls

As illustrated in Table 1, the cases and controls did not differ significantly with respect to the matching variables. The proportion of newly diagnosed hypertensive patients was similar between cases and controls. The mean duration of HTN in known hypertensive participants was significantly longer in participants with LVH than in those without LVH. Compared with patients without LVH, patients with LVH had significantly higher (p ˂ 0.05) BMI, WC, HC, and average 24-h systolic BP. A significantly larger proportion of sedentary persons was identified among patients with LVH (Table 1), with significantly increased measurements for RWT, E-wave deceleration time, E/e’ ratio (although within normal limits), triglyceridemia, AI, glycemia, HbA1c, insulinemia, HOMA-IR, IR, and hyperuricemia, compared with those in patients without LVH (Table 2). Conversely, the HDL-c level and E/A ratio were significantly reduced in patients with LVH compared with those in patients without LVH.

**Table 1:** General characteristics of Black hypertensive patients, stratified by the presence or absence of LVH

Characteristics	LVH + N = 88	LVH − N = 132	p-value
*Demographic characteristics*			
Age (years)	52.6 ± 10.6	50.3 ± 9.5	0.096
Sex			0.421
Male	52 (59.1)	81 (61.4)	
Female	36 (40.9)	51 (38.6)	
*Medical & social history*			
Known HTN	60 (68.2)	76 (57.6)	0.074
Duration of HTN (years)	5.0 (1.0–8.0)	4.0 (2.0–6.0)	0.014
ND HTN	28 (31.8)	56 (42.4)	0.149
Cigarette smoking	87 (98.9)	132 (100.0)	0.400
Alcohol intake	85 (96.6)	128 (97.0)	0.582
Menopause	14 (38.9)	27 (52.9)	0.141
*Anthropomorphic measurements*			
BMI (kg/m^2^)	32.6 ± 5.1	28.7 ± 4.3	< 0.001
WC (cm)	109.3 ± 13.2	99.3 ± 10.0	< 0.001
HC (cm)	112.7 ± 9.9	103.8 ± 9.2	< 0.001
Overweight	22 (25.0)	64 (48.5)	< 0.001
Total obesity	65 (73.9)	47 (35.6)	< 0.001
Abdominal obesity	34 (61.4)	43 (32.6)	< 0.001
*Lifestyle history*			
Sedentarity	71 (80.7)	52 (39.4)	< 0.001
Treatment history & examination findings			
Uncontrolled HTN	20 (22.7)	18 (13.6)	0.060
SBP (mmHg)	138.8 ± 7.8	133.4 ± 7.2	0.048
DBP (mmHg)	82.5 ± 8.7	79.9 ± 9.1	0.087
HR (bpm)	62.1 ± 13.5	70.0 ± 13.4	0.199

HTN = hypertension; ND HTN = newly diagnosed hypertension; WC = waist circumference; BMI = body mass index; HC = hip circumference; HR = heart rate; SBP = systolic blood pressure; DBP = diastolic blood pressure

**Table 2:** Echographic and biological characteristics of Black hypertensive patients, stratified by the presence or absence of LVH

Variables	LVH + n = 88	LVH − n = 132	P
*Echocardiographic measurements*			
LVED (mm)	46.5 ± 4.4	42.9 ± 4.1	** < 0.001**
IVS (mm)	12.7 ± 1.1	10.7 ± 1.5	** < 0.001**
PWT (mm)	12.5 ± 0.8	10.7 ± 1.5	** < 0.001**
SWT (mm)	25.2 ± 1.6	21.3 ± 2.9	** < 0.001**
LVEF (%)	63.8 ± 5.4	65.1 ± 4.9	0.062
LVM (g)	222.2 ± 38.4	156.8 ± 34.8	** < 0.001**
LVMIh (g/m^2,7^)	54.7 ± 8.4	37.6 ± 6.6	** < 0.001**
LVMIbsa (g/m^2^)	108.5 ± 15.7	79.7 ± 15.0	** < 0.001**
RWT	0.55 ± 0.1	0.50 ± 0.1	**0.001**
E (Cm/s)	0.85 ± 0.6	1.08 ± 0.6	**0.029**
E/A ratio	0.71 ± 0.2	0.99 ± 0.2	**0.034**
DT (ms)	215.8 ± 39.4	172.8 ± 37.7	** < 0.001**
E/e’ ratio	7.4 (4.9–7.5)	5.5 (4.5–7.0)	**˂ 0.001**
LAA (cm^2^)	17.3 ± 3.5	14.7 ± 2.7	**˂ 0.001**
SPAP (mmHg)	26.9 ± 3.1	26.0 ± 2.7	**0.019**
*Biological parameters*			
TC (mmol/L)	5.5 ± 1.0	5.4 ± 1.0	0.305
LDL-c (mmol/L)	3.8 ± 1.1	3.6 ± 1.1	**0.126**
Triglycerides (mmol/L)	1.25 ± 0.6	1.05 ± 0.6	**0.027**
HDL-c (mmol/L)	1.1 ± 0.3	1.3 ± 0.4	**0.003**
Glycemia (mmol/L)	6.3 ± 2.1	5.4 ± 1.6	** < 0.001**
HbA1C (%)	6.3 ± 1.6	5.9 ± 1.1	**0.016**
Insulinemia (mmol/L)	122.8 ± 43.1	72.7 ± 25.8	** < 0.001**
AI	5.2 ± 1.6	4.6 ± 1.8	**0.008**
HOMA-IR	2.36 ± 0.8	1.41 ± 0.6	**0.014**
Uric acid (mmol/L)	388.3 ± 98.4	352.9 ± 89.5	**0.007**
Creatinine (mmol/L)	84.7 ± 22.6	84.3 ± 16.2	0.854
Calcium (mmol/L)	2.30 ± 0.2	2.3 ± 0.2	0.105
Ionized calcium (mmol/L)	1.20 ± 0.11	1.22 ± 0.2	0.331
Phosphorus (mmol/L)	1.06 ± 0.2	1.09 ± 0.3	0.333
Dyslipidemia	75 (85.2)	98 (74.2)	**0.036**
High AI	45 (51.1)	48 (36.4)	**0.021**
T2DM	20 (22.7)	23 (17.4)	0.212
Hyperinsulinemia	8 (9.1)	11 (8.3)	0.514
IR	42 (47.7)	2 (1.5)	** < 0.001**
Hyperuricemia	29 (33.0)	22 (16.7)	**0.004**

LVED = left ventricular end-diastolic diameter; IVS = interventricular septal thickness; PWT = posterior wall thickness; SWT = sum of wall thickness; LVEF = left ventricular ejection fraction; LVM = left ventricular mass; LVMIh = left ventricular mass indexed to height^2,7^; LVMbsa = left ventricular mass indexed to body surface area; RWT = relative wall thickness; E = mitral E wave; E/A = ratio of peak early and late diastolic flow velocities; DT = deceleration time; e’ = mitral annular early diastolic velocity; LAA = left atrium area; SPAP = systolic pulmonary arterial pressure; TC = total cholesterol; LDL-c = low-density lipoprotein cholesterol; HDL-c = high-density lipoprotein; AI = atherogenicity index; HbA1C = glycated hemoglobin; T2DM = type 2 diabetes mellitus; HOMA-IR = homeostatic model assessment for insulin resistance; IR = insulin resistance

### Determinants of left ventricular mass

In the simple linear regression, a significant and positive relationship was identified between LVM and age, HTN duration, BMI, WC, glycemia, insulinemia, and HOMA-IR.

Nineteen percent (19%) of LVM variation was predicted by age, 31.3% by HTN duration, 44.4% by BMI, 42.5% by WC, 20% by glycemia, 44.8% by insulinemia, and 43.7%, by HOMA-IR (Table 3)

**Table 3:** Simple linear regression showing the determinants of left ventricular mass in Black patients with essential hypertension

Variables	R	Β	p
Age in years	0.190	0.22	**0.005**
HTN duration in years	0.313	0.57	** < 0.001**
BMI (kg/m^2^)	0.444	0.99	** < 0.001**
WC in cm	0.425	0.39	** < 0.001**
Glycemia (mmol/L)	0.201	1.19	**0.003**
Insulin (mmol/L)	0.448	0.12	** < 0.001**
HOMA-IR	0.437	5.80	** < 0.001**

HTN = hypertension; BMI = body mass index; WC = waist circumference; HOMA-IR = homeostatic model assessment for insulin resistance

In the multiple linear regression, the patient’s predicted LVM was equal to 0.56 (HTN duration) + 0.67 (BMI) + 0.08 (insulin levels) + 0.27 (HOMA-IR).

The HTN duration, BMI, insulin, and HOMA-IR predicted 68.3% of the patient’s LVM variations (Table 4).

**Table 4:** Multiple linear regression showing the determinants of left ventricular mass in Black patients with essential hypertension

Variables	LVM Ih		
	Β	SE	p
(Constant)	6.84	8.72	0.435
Age (years)	0.14	0.104	0.183
HTN duration	0.56	0.14	** < 0.001**
BMI (kg/m^2^)	0.67	0.23	**0.004**
WC (cm)	0.001	0.09	0.994
Glycemia (mmol/L)	0.06	0.46	0.903
Insulin (mmol/L)	0.08	0.04	**0.034**
HOMA-IR	0.27	1.81	**0.021**
	R^2^ = 0.683, overall p ˂ 0.001		

HTN = hypertension; BMI = body mass index; WC = waist circumference; HOMA-IR = homeostatic model assessment for insulin resistance

$${\text{Y }} = \, 0.{\text{56 X}}_{{1}} + \, 0.{\text{67 X}}_{{2}} + \, 0.0{\text{8 X}}_{{3}} + \, 0.{\text{27 X}}_{{4}} + { 6}.{84}$$

With Y = LVMIh; X_1_ = HTN duration; X_2_ = BMI; X_3_ = insulin; and X_4_ = HOMA-IR

### Determinants of LVH

In the univariate analysis, global obesity, abdominal obesity, sedentary status, AI, hyperuricemia, and IR were found to be significant predictors of LVH.

After multivariate adjustment, only total obesity and IR persisted as independent determinants of LVH. Obesity increased the risk of LVH three-fold [adjusted odds ratio (aOR) 2.8; 95% confidence interval (95% CI) 1.06–7.40, p = 0.038] and IR increased the risk of LVH eight-fold (aOR 8.4, 95% CI 3.7–15.7, p < 0.001, Table 5).

**Table 5:** Logistic regression analysis showing the determinants of LVH among Black hypertensive patients

Variables	Univariate analysis		Multivariate analysis	
	P	OR (95% CI)	p	aOR (95% CI)
*Total Obesity*				
No		1		1
Yes	0.000	5.1 (2.8–9.3)	**0.038**	2.8 (1.06–7.4)
*Abdominal Obesity*				
No		1		1
Yes	0.000	3.3 (1.9–5.8)	0.275	1.9 (0.6–6.3)
*Sedentary*				
No		1		1
Yes	0.000	6.4 (3.4–12.1)	0.123	1.9 (0.8–4.5)
*High AI*				
No		1		1
Yes	0.031	1.8 (1.06–3.2)	0.579	1.3 (0.6–2.9)
*Hyperuricemia*				
No		1		1
Yes	0.006	2.5 (1.3–4.7)	0.145	2.1 (0.8–5.4)
*IR*				
No		1		1
Yes	0.000	9.3 (3.8–25.5)	**˂0.001**	8.4 (3.7–15.7)

OR = odds ratio; aOR = adjusted odds ratio; AI = atherogenic index; IR = insulin resistance

## Discussion

In this study, four factors that could explain the bulk of the increase in LVM (68%) were established, including HTN duration, BMI, insulinemia, and HOMA-IR. However, only IR and total obesity emerged as independent determinants of LVH after multivariate analyses. We also observed that patients with LVH were more likely to describe themselves as sedentary, had higher obesity parameters, and more abnormalities in carbohydrate and lipid metabolism compared with those in patients without LVH. In addition, the patents with LVH also had significantly increased uric acid levels, AI, and E/e’ ratio and reduced E/A ratio and e’ values, with and a longer mitral E wave deceleration time.

Conflicting information exists regarding the involvement of IR in the development of LVH. Costa et al. [45] did not find any relationship between IR (with insulin measured during a glucose tolerance test) and LVM in a small sample of 35 non-obese, hypertensive Brazilian subjects. Galvan et al. [46], after adjusting for BP and BMI, also found that IR (with insulin sensitivity measured by the insulin clamp technique) was not an independent determinant of LVH in a small sample of 50 Italian non-diabetic subjects. These results in contrast to those found in the present study. Differences in the profile of the study population, the sample size, and the methods used to diagnose IR could explain these differences. In our study, HOMA-IR was used to diagnose IR. This method has the advantage of being easier to implement than the hyperinsulinemic-euglycemic glucose clamp, which is the current gold standard method for the determination of insulin sensitivity [47]. HOMA-IR has been the subject of numerous validation studies, which have demonstrated a satisfactory correlation between HOMA-IR and the gold standard method (r = 0.72 to 0.82, depending on the study), with no notable difference according to sex, age, weight, or diabetic or hypertensive status [48]. No established agreed HOMA-IR threshold for defining IR in the sub-Saharan Black African population. The cut-off of 2.5, which was used in the present study, has previously been used in Black Central African [49], African-American [50], European American [51], Caucasian [36], and Asian [52, 53] studies. However, our results agree with data obtained in populations other than Black sub-Saharan Africans. Sasson et al. [54] demonstrated a significant association between IR and LVH, which was independent of BP and BMI [59]. Lind et al. [55] also identified a similar association and demonstrated that hyperinsulinemia was responsible for 43% of the variation in LVM. In a recent prospective population study, Cauwenberghs et al. [56] found that basal IR/hyperinsulinemia and less favorable values measured at follow-up could predict left ventricular remodeling.

The pathophysiological arguments that can support this association are as follows. LVH is now recognized to be mediated not only by mechanical stress from pressure overload but also by various neurohormonal substances and metabolic abnormalities that independently exert trophic effects on cardiomyocytes and the extracellular matrix [22, 57]. This model has been substantiated by the high prevalence of LVH in normotensive type 2 diabetic individuals [58, 59]. In addition, IR, through multiple and complex mechanisms, has been shown to promote cardiomyocyte hypertrophy and matrix deposition, regardless of effects on systemic BP [60].

The downregulation of glucose transporter-4 expression in response to IR results in reduced transmembrane transport and mitochondrial glucose oxidation [61]. Under these conditions, energy metabolism depends on the oxidation of fatty acids for more than 90% of cellular energy requirements, increasing the plasma levels of fatty acids. The predominant oxidation of fatty acids and the reduction in the energy supply derived from glucose and pyruvates lead to the formation of end products of non-enzymatic glycation (AGEs or advanced glycation end-products), excess glycolytic compounds, and the increased synthesis of ceramide, all of which promote apoptosis. AGEs bind to specific receptors and activate protein kinase C, which stimulates the growth of median connective tissue and the synthesis of collagen, promoting the development of interstitial fibrosis. Additionally, IR and the increased mitochondrial influx of fatty acids cause the overproduction of superoxide ions, which are involved in the genesis of hypertrophy, fibrosis, and left ventricular dysfunction [62].

The association between the HTN duration and LVH has been highlighted in several previous studies. In the Democratic Republic of Congo, Lepira et al. [63] demonstrated that the HTN duration could predict the occurrence of electrical LVH. This association accounts for the influence of the duration of myocardial exposure to chronic barometric overload, which is represented by HTN.

In the present study, compared with patients without LVH, we found that hypertensive participants with LVH had a lower E/A ratio and a longer deceleration time, which indicated abnormalities in relaxation [41, 42, 64], associated with normal LVFP, as evidenced by a normal E/e’ ratio (˂8) [43], with an almost-normal LAA. The presence of an isolated relaxation abnormality, without an associated impact on filling pressures, is thought to be due to the relatively short HTN (5 years and 4 years in participants with LVH and patients without LVH, respectively). Diastolic dysfunction is a consequence of both IR [65, 66] and LVH, and the underlying myocardial fibrosis [42, 67–70]. In addition, the mitochondrial dysfunction that accompanies the IR state is thought to play a role in both LVH and diastolic dysfunction [71]. However, this association remains under debate. On the one hand, a certain degree of diastolic dysfunction exists in hypertensive patients long before they develop LVH [72]; on the other hand, the regression of LVH after antihypertensive treatment does not necessarily result in the normalization of diastolic function [74].

Our hypertensive patients with LVH were often sedentary, with higher obesity parameters, and more abnormalities in carbohydrate and lipid metabolism than the matched patients without LVH. The relationship between a sedentary lifestyle and LVM remains controversial. Gibbs et al. [75] observed relationships between a sedentary lifestyle, obesity, and increased LVM in Caucasian adults but not in Black populations. In a previous analysis, we assessed this association in both a sub-Saharan Black population and a White Maghrebi population and found that a sedentary lifestyle was associated with a lower LVM in the White Maghrebi population but not in the sub-Saharan Black population [76]. Similarly, in the present study of sub-Saharan Black patients, although a larger proportion of the patients with LVH were sedentary, no significant association was found between a sedentary lifestyle and LVM. Potential qualitative differences might exist in the cardiovascular consequences of sedentary behaviors among various populations.

The association between obesity and LVH appears to be a common finding. However, some divergence exists with regard to the concentric or eccentric geometry patterns of LVH among obese hypertensive patients. Some authors have found a predominance of eccentric geometry [77], whereas others, including our group, have found a predominance of concentric geometry [78, 79]. Concentric geometry is more often attributed to pressure overload, whereas eccentric geometry is attributed to volume overload [80]. The co-occurrence of HTN, which is associated with pressure overload, and obesity, which is a condition of volume overload, results in a phenotype that is determined by the predominance of one over the other. This explains the divergent results in the literature based on the study population. Furthermore, an initially concentric geometry can evolve over time towards an eccentric geometry.

The combination of sedentary behavior and obesity is essentially characterized by a chronic caloric excess. Experimental research has indicated that prolonged and uninterrupted sitting sessions lead to increased blood levels of insulin and glucose. Obesity is linked to IR via complex mechanisms, including inflammation due to the accumulation of lipids, the inhibitory effects of fatty acid oxidation on glucose oxidation, and the secretion of adipocytokines, which have all been associated with the development of local and systemic IR [81]. Therefore, IR might bridge the gap between a sedentary lifestyle/obesity and LVH.

Significantly higher uric acid levels were found in hypertensives with LVH than in those without LVH, which is in agreement with previous studies that have reported that hypertensives with LVH have higher uric acid levels [82]. A causal link has been suggested because the normalization of uric acid levels using a hypouricemic treatment resulted in the reduced LVM [83, 84]. Several mechanisms could be used to explain the increase in LVM associated with hyperuricemia, including the systemic inflammatory response, oxidative stress [85, 86], the activity of the renin–angiotensin–aldosterone system [87], endothelial dysfunction [88], and the expression of endothelin-1 in cardiac fibroblasts, which promotes interstitial fibrosis in the myocardium [89]. Furthermore, some indirect effects of hyperuricemia, such as increased BP, a parallel decrease in the glomerular filtration rate, the deterioration of adhesion, platelet aggregation, and increased aortic stiffness, could further contribute to the development of LVH [90].

Finally, this study identified a higher AI value in hypertensive participants with LVH than in those without LVH, which suggested an increase in the risk of coronary events, which aligns with a previous study that established LVH as a risk factor for coronary heart disease associated mortality [91].

### Study limitations

Our study must be interpreted within the context of its potential limitations and strengths. First, echocardiographic measurements are prone to measurement errors due to signal noise, acoustic artifacts, and angle dependency. In addition, the intraobserver variability associated with the performance of transthoracic 2D echocardiography is not as good as the real-time 3D technique [28, 92]; however, in the present study, echocardiography was performed by an experienced cardiologist with post-graduate training in cardiac imaging. Second, the case–control design we used precluded the assessment of cause-effect relationships. Third, the in-hospital and monocentric design makes it risky to extrapolate the results to all sub-Saharan Black hypertensive patients. Our study covers a gap because, to the best of our knowledge, this study represents the first description of the association between IR and LVH in Black sub-Saharan African hypertensive patients.

## Conclusions

Our results showed direct and significant associations between the HTN duration, BMI, insulinemia, and HOMA-IR and LVM. The multivariate analysis revealed IR and obesity as independent determinants of LVH in HTN. These results indicated that in addition to hemodynamic factors related to high BP, changes in LVM in hypertensive patients might also be mediated by IR. The early detection and effective management of IR should be considered in all hypertensive patients to prevent or delay the development of LVH and its consequences. In addition, these results should stimulate further research to assess the efficacy and safety of pharmacological and nonpharmacological insulin sensitization measures on IR in hypertensive patients, even those who are classified as non-diabetic.

A prospective Black sub-Saharan population-based study with serial imaging remains essential to better understand subclinical LV deterioration over time and to confirm the role played by IR in Black sub-Saharan hypertensive patients.

## Data Availability

Because the consent given by study participants did not include data sharing with third parties, anonymized data can be made available to investigators for analysis on reasonable request to the corresponding author.

## Abbreviations

ABPM: Ambulatory blood pressure monitoring
AI: Atherogenicity index
BMI: Body mass index
BSA: Body surface area
DBP: Diastolic blood pressure.
DT: Deceleration time
E/A: Ratio of peak early and late diastolic flow velocities
E: Mitral E wave
HbA1C: Glycated hemoglobin
HC: Hip circumference
HDL-c: High-density lipoprotein cholesterol
HMOD: Hypertension-mediated organ damage
HOMA-IR: Homeostatic model assessment for insulin resistance
HR: Heart rate
HTN: Hypertension
IR: Insulin resistance.
IVS: Interventricular septal thickness
LAA: Left atrium area
LDL-c: Low density lipoprotein cholesterol
LVED: Left ventricular end-diastolic diameter
LVEF: Left ventricular ejection fraction
LVM: Left ventricular mass
LVMIbs: Left ventricular mass indexed to body surface area
LVMIh: Left ventricular mass indexed to height ^2,7^
ND HTN: Newly-diagnosed hypertension
PWT: Posterior wall thickness
RWT: Relative wall thickness
SBP: Systolic blood pressure
SPAP: Systolic pulmonary arterial pressure
SWT: Sum of wall thickness
T2DM: Type 2 diabetes mellitus
TC: Total cholesterol
WC: Waist circumference

## References

[1] Bigazzi R, Bianchi S, Buoncristiani E, Campese VM (2008). Increased cardiovascular events in hypertensive patients with insulin resistance: a 13-year follow-up. Nutr Metab Cardiovasc Dis.

[2] Williams B, Mancia G, Spiering W, Agabiti Rosei E, Azizi M, Burnier M (2018). 2018 ESC/ESH guidelines for the management of arterial hypertension. Eur Heart J.

[3] Petersen MC, Shulman GI (2018). Mechanisms of insulin action and insulin resistance. Physiol Rev.

[4] Shen SW, Reaven GM, Farquhar JW (1970). Comparison of impedance to insulin-mediated glucose uptake in normal subjects and in subjects with latent diabetes. J Clin Invest.

[5] Ginsberg H, Kimmerling G, Olefsky JM, Reaven GM (1975). Demonstration of insulin resistance in untreated adult onset diabetic subjects with fasting hyperglycemia. J Clin Invest.

[6] Olefsky J, Farquhar JW, Reaven G (1973). Relationship between fasting plasma insulin level and resistance to insulin-mediated glucose uptake in normal and diabetic subjects. Diabetes.

[7] Fuh MM, Shieh SM, Wu DA, Chen YD, Reaven GM (1987). Abnormalities of carbohydrate and lipid metabolism in patients with hypertension. Arch Intern Med.

[8] Zavaroni I, Coruzzi P, Bonini L, Mossini GL, Musiari L, Gasparini P (1995). Association between salt sensitivity and insulin concentrations in patients with hypertension. Am J Hypertens.

[9] Reaven GM, Lithell H, Landsberg L (1996). Hypertension and associated metabolic abnormalities–the role of insulin resistance and the sympathoadrenal system. N Engl J Med.

[10] Abbasi F, McLaughlin T, Lamendola C, Lipinska I, Tofler G, Reaven GM (1999). Comparison of plasminogen activator inhibitor-1 concentration in insulin-resistant versus insulin-sensitive healthy women. Arterioscler Thromb Vasc Biol.

[11] Facchini F, Chen YD, Hollenbeck CB, Reaven GM (1991). Relationship between resistance to insulin-mediated glucose uptake, urinary uric acid clearance, and plasma uric acid concentration. JAMA.

[12] Chen YD, Swami S, Skowronski R, Coulston A, Reaven GM (1993). Differences in postprandial lipemia between patients with normal glucose tolerance and noninsulin-dependent diabetes mellitus. J Clin Endocrinol Metab.

[13] Reaven GM, Lerner RL, Stern MP, Farquhar JW (1967). Role of insulin in endogenous hypertriglyceridemia. J Clin Invest.

[14] Golay A, Zech L, Shi MZ, Chiou YA, Reaven GM, Chen YD (1987). High density lipoprotein (HDL) metabolism in noninsulin-dependent diabetes mellitus: measurement of HDL turnover using tritiated HDL. J Clin Endocrinol Metab.

[15] Reaven GM, Chen YD, Jeppesen J, Maheux P, Krauss RM (1993). Insulin resistance and hyperinsulinemia in individuals with small, dense low density lipoprotein particles. J Clin Invest.

[16] Stewart MH, Lavie CJ, Shah S, Englert J, Gilliland Y, Qamruddin S (2018). Prognostic implications of left ventricular hypertrophy. Prog Cardiovasc Dis.

[17] Bombelli M, Facchetti R, Carugo S, Madotto F, Arenare F, Quarti-Trevano F (2009). Left ventricular hypertrophy increases cardiovascular risk independently of in-office and out-of-office blood pressure values. J Hypertens.

[18] Gardin JM, McClelland R, Kitzman D, Lima JA, Bommer W, Klopfenstein HS (2001). M-mode echocardiographic predictors of six- to seven-year incidence of coronary heart disease, stroke, congestive heart failure, and mortality in an elderly cohort (the Cardiovascular Health Study). Am J Cardiol.

[19] Desai CS, Bartz TM, Gottdiener JS, Lloyd-Jones DM, Gardin JM (2016). Usefulness of left ventricular mass and geometry for determining 10-year prediction of cardiovascular disease in adults aged >65 years (from the cardiovascular health study). Am J Cardiol.

[20] Yu W, Chen C, Fu Y, Wang X, Wang W (2010). Insulin signaling: a possible pathogenesis of cardiac hypertrophy. Cardiovasc Ther.

[21] Sharma P, Middelberg RP, Andrew T, Johnson MR, Christley H, Brown MJ (2006). Heritability of left ventricular mass in a large cohort of twins. J Hypertens.

[22] Chien KL, Hsu HC, Su TC, Chen MF, Lee YT (2006). Heritability and major gene effects on left ventricular mass in the Chinese population: a family study. BMC Cardiovasc Disord.

[23] Harshfield GA, Grim CE, Hwang C, Savage DD, Anderson SJ (1990). Genetic and environmental influences on echocardiographically determined left ventricular mass in Black twins. Am J Hypertens.

[24] Lorell BH, Carabello BA (2000). Left ventricular hypertrophy: pathogenesis, detection, and prognosis. Circulation.

[25] Devereux RB, Roman MJ, Paranicas M, O’Grady MJ, Lee ET, Welty TK (2000). Impact of diabetes on cardiac structure and function: the strong heart study. Circulation.

[26] Ilercil A, Devereux RB, Roman MJ, Paranicas M, O’Grady MJ, Welty TK (2001). Relationship of impaired glucose tolerance to left ventricular structure and function: the strong heart study. Am Heart J.

[27] Du Bois D, Du Bois EF (1989). A formula to estimate the approximate surface area if height and weight be known. 1916. Nutrition.

[28] Lang RM, Badano LP, Mor-Avi V, Afilalo J, Armstrong A, Ernande L (2015). Recommendations for cardiac chamber quantification by echocardiography in adults: an update from the American Society of Echocardiography and the European Association of Cardiovascular Imaging. Eur Heart J Cardiovasc Imaging.

[29] Nagueh SF, Smiseth OA, Appleton CP, Byrd BF, Dokainish H, Edvardsen T (2016). Recommendations for the evaluation of left ventricular diastolic function by echocardiography: an update from the American society of echocardiography and the European association of cardiovascular imaging. J Am Soc Echocardiogr.

[30] Chau JY, Grunseit AC, Chey T, Stamatakis E, Brown WJ, Matthews CE (2013). Daily sitting time and all-cause mortality: a meta-analysis. PLoS ONE.

[31] Ryan H, Trosclair A, Gfroerer J (2012). Adult current smoking: differences in definitions and prevalence estimates–NHIS and NSDUH, 2008. J Environ Public Health.

[32] Moos RH, Schutte KK, Brennan PL, Moos BS (2009). Older adults’ alcohol consumption and late-life drinking problems: a 20-year perspective. Addiction.

[33] GBD 2015 Obesity Collaborators, Afshin A, Forouzanfar MH, Reitsma MB, Sur P, Estep K, et al.: Health effects of overweight and obesity in 195 countries over 25 years. N Engl J Med. 2017;377:13–27. 10.1056/NEJMoa1614362 PMC5477817 28604169

[34] O’Brien E, White WB, Parati G, Dolan E (2018). Ambulatory blood pressure monitoring in the 21st century. J Clin Hypertens (Greenwich).

[35] d’Emden MC, Shaw JE, Jones GR, Cheung NW (2015). Guidance concerning the use of glycated haemoglobin (HbA1c) for the diagnosis of diabetes mellitus. Med J Aust.

[36] Ramos-Lopez O, Riezu-Boj JI, Milagro FI, Cuervo M, Goni L, Martinez JA (2019). Interplay of an obesity-based genetic risk score with dietary and endocrine factors on insulin resistance. Nutrients.

[37] Wu L, Parhofer KG (2014). Diabetic dyslipidemia. Metabolism.

[38] Elshazly MB, Nicholls SJ, Nissen SE, St John J, Martin SS, Jones SR (2016). Implications of total to high-density lipoprotein cholesterol ratio discordance with alternative lipid parameters for coronary atheroma progression and cardiovascular events. Am J Cardiol.

[39] Kerola T, Kauppi J, Sares-Jaske L, Anttonen O, Junttila MJ, Huikuri HV (2019). Long-term prognostic impact of hyperuricemia in community. Scand J Clin Lab Invest.

[40] Oktay AA, Lavie CJ, Milani RV, Ventura HO, Gilliland YE, Shah S (2016). Current perspectives on left ventricular geometry in systemic hypertension. Prog Cardiovasc Dis.

[41] Galderisi M, Cosyns B, Edvardsen T, Cardim N, Delgado V, Di Salvo G (2017). Standardization of adult transthoracic echocardiography reporting in agreement with recent chamber quantification, diastolic function, and heart valve disease recommendations: an expert consensus document of the European Association of Cardiovascular Imaging. Eur Heart J Cardiovasc Imaging.

[42] Nagueh SF (2020). Left ventricular diastolic function: understanding pathophysiology, diagnosis, and prognosis with echocardiography. JACC Cardiovasc Imaging.

[43] Sharifov OF, Schiros CG, Aban I, Denney TS, Gupta H (2016). Diagnostic accuracy of tissue doppler index e/e’ for evaluating left ventricular filling pressure and diastolic dysfunction/heart failure with preserved ejection fraction: a systematic review and meta-analysis. J Am Heart Assoc.

[44] Lang RM, Badano LP, Mor-Avi V, Afilalo J, Armstrong A, Ernande L (2015). Recommendations for cardiac chamber quantification by echocardiography in adults: an update from the American Society of Echocardiography and the European Association of Cardiovascular Imaging. J Am Soc Echocardiogr.

[45] Costa CH, Batista MC, Moises VA, Kohlmann NB, Ribeiro AB, Zanella MT (1995). Serum insulin levels, 24-hour blood pressure profile, and left ventricular mass in non-obese hypertensive patients. Hypertension.

[46] Galvan AQ, Galetta F, Natali A, Muscelli E, Sironi AM, Cini G (2000). Insulin resistance and hyperinsulinemia: no independent relation to left ventricular mass in humans. Circulation.

[47] Singh B, Saxena A (2010). Surrogate markers of insulin resistance: a review. World J Diabetes.

[48] Bonora E, Targher G, Alberiche M, Bonadonna RC, Saggiani F, Zenere MB (2000). Homeostasis model assessment closely mirrors the glucose clamp technique in the assessment of insulin sensitivity: studies in subjects with various degrees of glucose tolerance and insulin sensitivity. Diabetes Care.

[49] On’kin J, Longo-Mbenza B, Tchokonte-Nana V, Okwe AN, Kabangu NK. Hyperbolic relation between beta-cell function and insulin sensitivity for type 2 diabetes mellitus, malaria, influenza, Helicobacter pylori, Chlamydia pneumoniae, and hepatitis C virus infection-induced inflammation/oxidative stress and temporary insulin resistance in Central Africans. Turk J Med Sci. 2017;47:1834–41. 10.3906/sag-1608-48 29306246

[50] Vardeny O, Gupta DK, Claggett B, Burke S, Shah A, Loehr L (2013). Insulin resistance and incident heart failure the ARIC study (Atherosclerosis Risk in Communities). JACC Heart Fail.

[51] Owei I, Umekwe N, Provo C, Wan J, Dagogo-Jack S (2017). Insulin-sensitive and insulin-resistant obese and non-obese phenotypes: role in prediction of incident pre-diabetes in a longitudinal biracial cohort. BMJ Open Diabetes Res Care.

[52] Yamada C, Mitsuhashi T, Hiratsuka N, Inabe F, Araida N, Takahashi E (2011). Optimal reference interval for homeostasis model assessment of insulin resistance in a Japanese population. J Diabetes Investig.

[53] Singh Y, Garg MK, Tandon N, Marwaha RK (2013). A study of insulin resistance by HOMA-IR and its cut-off value to identify metabolic syndrome in urban Indian adolescents. J Clin Res Pediatr Endocrinol.

[54] Sasson Z, Rasooly Y, Bhesania T, Rasooly I (1993). Insulin resistance is an important determinant of left ventricular mass in the obese. Circulation.

[55] Lind L, Andersson PE, Andren B, Hanni A, Lithell HO (1995). Left ventricular hypertrophy in hypertension is associated with the insulin resistance metabolic syndrome. J Hypertens.

[56] Cauwenberghs N, Knez J, Thijs L, Haddad F, Vanassche T, Yang WY (2018). Relation of insulin resistance to longitudinal changes in left ventricular structure and function in a general population. J Am Heart Assoc.

[57] Nwabuo CC, Vasan RS (2020). Pathophysiology of hypertensive heart disease: beyond left ventricular hypertrophy. Curr Hypertens Rep.

[58] Sato A, Tarnow L, Nielsen FS, Knudsen E, Parving HH (2005). Left ventricular hypertrophy in normoalbuminuric type 2 diabetic patients not taking antihypertensive treatment. QJM.

[59] Santra S, Basu AK, Roychowdhury P, Banerjee R, Singhania P, Singh S (2011). Comparison of left ventricular mass in normotensive type 2 diabetes mellitus patients with that in the non-diabetic population. J Cardiovasc Dis Res.

[60] Velagaleti RS, Gona P, Chuang ML, Salton CJ, Fox CS, Blease SJ (2010). Relations of insulin resistance and glycemic abnormalities to cardiovascular magnetic resonance measures of cardiac structure and function: the Framingham Heart Study. Circ Cardiovasc Imaging.

[61] Yilmaz S, Canpolat U, Aydogdu S, Abboud HE (2015). Diabetic cardiomyopathy; summary of 41 years. Korean Circ J.

[62] Letonja M, Petrovic D (2014). Is diabetic cardiomyopathy a specific entity?. World J Cardiol.

[63] Lepira FB, Kayembe PK, M’Buyamba-Kabangu JR, Nseka MN (2006). Clinical correlates of left ventricular hypertrophy in Black patients with arterial hypertension. Cardiovasc J S Afr.

[64] Silbiger JJ (2019). Pathophysiology and echocardiographic diagnosis of left ventricular diastolic dysfunction. J Am Soc Echocardiogr.

[65] Devereux RB, de Simone G, Palmieri V, Oberman A, Hopkins P, Kitzman DW (2002). Relation of insulin to left ventricular geometry and function in African American and white hypertensive adults: the HyperGEN study. Am J Hypertens.

[66] Olszanecka A, Dragan A, Kawecka-Jaszcz K, Fedak D, Czarnecka D (2017). Relationships of insulin-like growth factor-1, its binding proteins, and cardiometabolic risk in hypertensive perimenopausal women. Metabolism.

[67] Lopez B, Gonzalez A, Diez J (2010). Circulating biomarkers of collagen metabolism in cardiac diseases. Circulation.

[68] Ihm SH, Youn HJ, Shin DI, Jang SW, Park CS, Kim PJ (2007). Serum carboxy-terminal propeptide of type I procollagen (PIP) is a marker of diastolic dysfunction in patients with early type 2 diabetes mellitus. Int J Cardiol.

[69] Wachtell K, Smith G, Gerdts E, Dahlof B, Nieminen MS, Papademetriou V, et al. Left ventricular filling patterns in patients with systemic hypertension and left ventricular hypertrophy (the LIFE study). Losartan intervention for endpoint. Am J Cardiol. 2000;85:466–72. 10.1016/s0002-9149(99)00773-0 10728952

[70] Spinale FG (2002). Bioactive peptide signaling within the myocardial interstitium and the matrix metalloproteinases. Circ Res.

[71] Lahera V, de Las HN, Lopez-Farre A, Manucha W, Ferder L (2017). Role of mitochondrial dysfunction in hypertension and obesity. Curr Hypertens Rep.

[72] El Saiedi SA, Mira MF, Sharaf SA, Al Musaddar MM, El Kaffas RMH, AbdelMassih AF (2018). Left ventricular diastolic dysfunction without left ventricular hypertrophy in obese children and adolescents: a Tissue Doppler Imaging and Cardiac Troponin I Study. Cardiol Young.

[73] Solomon SD, Appelbaum E, Manning WJ, Verma A, Berglund T, Lukashevich V (2009). Effect of the direct Renin inhibitor aliskiren, the Angiotensin receptor blocker losartan, or both on left ventricular mass in patients with hypertension and left ventricular hypertrophy. Circulation.

[74] Wachtell K, Bella JN, Rokkedal J, Palmieri V, Papademetriou V, Dahlof B (2002). Change in diastolic left ventricular filling after one year of antihypertensive treatment: the losartan intervention for endpoint reduction in hypertension (LIFE) study. Circulation.

[75] Gibbs BB, Reis JP, Schelbert EB, Craft LL, Sidney S, Lima J (2014). Sedentary screen time and left ventricular structure and function: the CARDIA study. Med Sci Sports Exerc.

[76] Annis C, Phanzu BK, Sidibe M, El Hattaoui M, Dounia B, Kabangu J-RMb, et al. The influence of ethnicity in the relationship between sedentary screen time and left ventricular mass: insights from the MAG-SALVAGES. World J Cardiovasc Dis. 2017;7:11–23.

[77] Fox E, Taylor H, Andrew M, Han H, Mohamed E, Garrison R (2004). Body mass index and blood pressure influences on left ventricular mass and geometry in African Americans: the atherosclerotic risk in communities (ARIC) study. Hypertension.

[78] Avelar E, Cloward TV, Walker JM, Farney RJ, Strong M, Pendleton RC (2007). Left ventricular hypertrophy in severe obesity: interactions among blood pressure, nocturnal hypoxemia, and body mass. Hypertension.

[79] Woodiwiss AJ, Libhaber CD, Majane OH, Libhaber E, Maseko M, Norton GR (2008). Obesity promotes left ventricular concentric rather than eccentric geometric remodeling and hypertrophy independent of blood pressure. Am J Hypertens.

[80] Grossman W, Paulus WJ (2013). Myocardial stress and hypertrophy: a complex interface between biophysics and cardiac remodeling. J Clin Invest.

[81] Abel ED, O’Shea KM, Ramasamy R (2012). Insulin resistance: metabolic mechanisms and consequences in the heart. Arterioscler Thromb Vasc Biol.

[82] Catena C, Colussi G, Capobianco F, Brosolo G, Sechi LA (2014). Uricaemia and left ventricular mass in hypertensive patients. Eur J Clin Invest.

[83] Gingles CR, Symon R, Gandy SJ, Struthers AD, Houston G, MacDonald TM (2019). Allopurinol treatment adversely impacts left ventricular mass regression in patients with well-controlled hypertension. J Hypertens.

[84] Szwejkowski BR, Gandy SJ, Rekhraj S, Houston JG, Lang CC, Morris AD (2013). Allopurinol reduces left ventricular mass in patients with type 2 diabetes and left ventricular hypertrophy. J Am Coll Cardiol.

[85] Takimoto E, Kass DA (2007). Role of oxidative stress in cardiac hypertrophy and remodeling. Hypertension.

[86] Takimoto E, Champion HC, Li M, Ren S, Rodriguez ER, Tavazzi B (2005). Oxidant stress from nitric oxide synthase-3 uncoupling stimulates cardiac pathologic remodeling from chronic pressure load. J Clin Invest.

[87] Corry DB, Eslami P, Yamamoto K, Nyby MD, Makino H, Tuck ML (2008). Uric acid stimulates vascular smooth muscle cell proliferation and oxidative stress via the vascular renin-angiotensin system. J Hypertens.

[88] Yu MA, Sanchez-Lozada LG, Johnson RJ, Kang DH (2010). Oxidative stress with an activation of the renin-angiotensin system in human vascular endothelial cells as a novel mechanism of uric acid-induced endothelial dysfunction. J Hypertens.

[89] Cheng TH, Lin JW, Chao HH, Chen YL, Chen CH, Chan P (2010). Uric acid activates extracellular signal-regulated kinases and thereafter endothelin-1 expression in rat cardiac fibroblasts. Int J Cardiol.

[90] Ramirez-Sandoval JC, Sanchez-Lozada LG, Madero M (2017). Uric acid, vascular stiffness, and chronic kidney disease: is there a link?. Blood Purif.

[91] Brown DW, Giles WH, Croft JB (2000). Left ventricular hypertrophy as a predictor of coronary heart disease mortality and the effect of hypertension. Am Heart J.

[92] Mor-Avi V, Sugeng L, Weinert L, MacEneaney P, Caiani EG, Koch R (2004). Fast measurement of left ventricular mass with real-time three-dimensional echocardiography: comparison with magnetic resonance imaging. Circulation.

